# Genetic analysis of patients with Fuchs endothelial corneal dystrophy in India

**DOI:** 10.1186/1471-2415-10-3

**Published:** 2010-02-10

**Authors:** Boomiraj Hemadevi, Muthiah Srinivasan, Jambulingam Arunkumar, Namperumalsamy V Prajna, Periasamy Sundaresan

**Affiliations:** 1 Department of Genetics, Dr G Venkataswamy Eye Research Institute (Aravind Medical Research Foundation), #1 Anna nagar, Madurai, Tamilnadu, India; 2 Cornea Services, Aravind Eye Hospital, Madurai, Tamilnadu, India

## Abstract

**Background:**

Mutations in *COL8A2 *gene which encodes the collagen alpha-2 (VIII) chain have been identified in both familial and sporadic cases of Fuchs endothelial corneal dystrophy (FECD). Heterozygous mutations in the *SLC4A11 *gene are also known to cause late-onset FECD. Therefore we screened for *COL8A2*, *SLC4A11 *gene variants in Indian FECD patients.

**Methods:**

Eighty patients with clinically diagnosed FECD and 100 age matched normal individuals were recruited. Genomic DNA was isolated from peripheral blood leukocytes. Mutations in *COL8A2*, *SLC4A11 *coding regions were screened using bi-directional sequencing. Fischer's exact test or Pearson's chi squared test were used to predict the statistical association of genotypes with the phenotype.

**Results:**

Screening of *COL8A2 *gene revealed 2 novel c.1610G>A, c.1643A>G and 3 reported variations c.112G>A, c.464G>A and c.1485G>A. In *SLC4A11 *gene, novel c.1659C>T, c.1974C>T and reported c.405G>A, c.481A>C and c.639G>A variants were identified. However all the variations in both the genes were also present in unaffected controls.

**Conclusions:**

This is the first study analysing *COL8A2 *gene in Indian patients with FECD. No pathogenic mutations were identified in *COL8A2*. Merely silent changes, which showed statistically insignificant association with FECD, were identified in the screening of *SLC4A11 *gene. These results suggest that *COL8A2*, *SLC4A11 *genes may not be responsible for FECD in patients examined in this study.

## Background

The corneal endothelial dystrophies comprising Fuchs endothelial corneal dystrophy (FECD; MIM#s 136800 and 610158), posterior polymorphous corneal dystrophy (PPCD; MIM#s 122000, 609140 and 609141) and congenital hereditary endothelial dystrophy (CHED; MIM121700 and 217700) are thought to represent defects of neural crest terminal differentiation [1]. FECD is a degenerative, bilateral, often asymmetric and slowly progressive disorder [2]. It is characterized by a pleomorphic, attenuated, dysfunctional and degenerated corneal endothelium and the progressive formation of microscopic, refractile, posterior excrescences in the Descemet membrane (a collagen rich basal lamina) secreted by corneal endothelium, clinically known as corneal guttae [3]. Due to endothelial dysfunction and loss of cells with FECD progression leads to corneal decompensation and impaired vision [4].

FECD may show familial clustering but it is usually a sporadic condition [5,6]. It occurs predominantly in females compared to the males [2,7]. Corneal grafting is the only means of restoring vision in advanced cases, however there is always the risk for allograft rejection and requires lifelong follow up.

The genotypic approach will provide better understanding, identification of the underlying genetic defects and in future enhance the possibility of medical intervention using conventional pharmacological approaches or gene therapy. Family based studies have mapped late-onset FECD susceptibility loci to 13ptel-13q12.13 [8] and 18q21.2-q21.32 [9]. Genome wide linkage analysis of 22 families with FECD identified potential linkage regions on chromosomes 1, 7, 15, 17, and X [10]. Recently FECD is linked to a novel locus on 5q33.1-q35.2 [11]. Mutations in the *COL8A2 *gene located on 1p34.3 have been described in patients with FECD [12-16]. This gene encodes for the collagen alpha- 2 (VIII) chain, a major component of Descemet membrane. This protein is found in the posterior banded layer (PBL) of patients with FECD [17,18].

Heterozygous mutations in the *SLC4A11 *gene are also known to be associated with the late-onset FECD [19]. The *SLC4A11 *gene, which codes for sodium bicarbonate transporter-like protein 11 have been previously associated with autosomal recessive congenital hereditary endothelial dystrophy (CHED2) that also arises by the primary defect in the corneal endothelium [20].

Susceptibility of genes to mutations can vary in different ethnicities and also in view of the limited information on the genetics of FECD from India, we undertook this study. We screened for mutation in the *COL8A2 *and *SLC4A11 *genes to determine whether these genes are responsible for causing FECD in Indian population. We report for the first time, the results of mutation analysis of the *COL8A2 *gene in 80 unrelated patients with early onset & late onset FECD. A study by Vithana et al., screened 25 Indian FECD patients for *SLC4A11 *mutations [19], this is the first report of analysing *SLC4A11 *gene in a larger series of Indian patients having FECD.

## Methods

### Patients

All the patients and normal controls screened were recruited from Aravind Eye Hospital (A tertiary eye care centre), Madurai, Tamilnadu, India. Eighty patients studied were sporadic cases of Indian ancestry. One hundred control individuals without any eye disease were enlisted. The study protocol had the approval of Institutional Review Board of Aravind Eye Hospital. Informed consent was obtained from each individual and all studies were performed according to the tenets of the Declaration of Helsinki. All patients underwent a complete ophthalmic evaluation including a detailed medical and family history. The presence of guttae was identified using the slit lamp and confirmed by specular microscopy. Histopathological examination of corneas collected from the patients who underwent penetrating keratoplasty (PKP) was also done.

### Mutation analysis

DNA was isolated from peripheral blood lymphocytes using the protocol described by Miller et al., (1988) [21]. Polymerase chain reaction (PCR) was used to amplify the 2 exons of *COL8A2 *(NM_005202) using 16 sets of oligonucleotide primer pairs and PCR conditions were followed as previously described in the literature [12]. For mutational analysis of *SLC4A11 *(NM_032034) gene, all the coding exons of *SLC4A11 *and their flanking splice junctions were amplified by means of PCR using the primers reported elsewhere [20]. The PCR products were purified using a gel extraction kit (Amersham Biosciences, Piscataway, NJ), and bi-directional sequencing was performed using an ABI 3130 sequencer (Applied Biosystems, Foster City, CA).

### Statistical analysis

Data was analysed using Stata 8 (Stata, college station, TX). Statistical differences in allele and genotype frequencies between the case and the control groups were determined using Pearson's chi-squared test or by Fischer's exact test respectively.

### Prediction of splice site alteration

To identify whether the variants found are causing any splice site alteration, a program for recognizing the splice sites was used http://www.fruitfly.org/seq_tools/splice.html.

## Results

Eighty unrelated patients with a diagnosis of either early or late-onset FECD were enrolled in the study. All of them were sporadic cases and no family history of disease was observed. Among the 80 patients, 27 were men and 53 were women. Age of onset of symptoms was between 17 to 76 years. The probands' onset of symptoms within 40 years of age was considered as early onset and above 40 years as late onset. Fifteen cases came under the early onset category in which 5 were males and 10 were females. Their age ranged from 26 to 40 years with an average of 33.6 years (Standard Deviation (SD) 5.1). The age of 65 late onset cases ranged from 42 to 76 years with an average range of 55 years (SD 8.3) including 20 males and 45 females. The 100 unrelated controls age range was 25 to 70 years with an average of 50 years (SD 12.42). Among the 80 FECD patients 37 patients underwent PKP (bilateral- 11, unilateral- 26), the age at surgery was between 36 to 77 years and all the cases were confirmed to have FECD by histopathological examination. Epithelial, stromal edema and thickened Descemet membrane was seen in all the buttons including the presence of guttae in longstanding cases.

**Figure 1:**
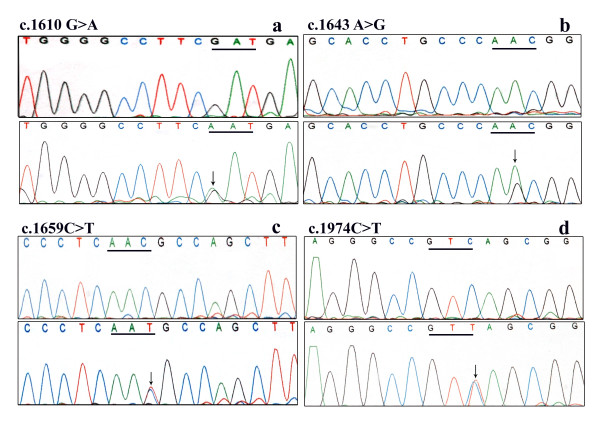
**Sequence chromatograms of the FECD patients showing the variants along with the controls**. a, b. Novel variants identified in the *COL8A2 *gene. c, d. Novel variants identified in *SLC4A11 *gene. The underline marks the mutated codon. The arrow indicates heterozygous state of the sequence.

Screening of the *COL8A2 *gene in the affected patients did not reveal any of the previously identified pathogenic mutations associated with FECD [12,13,15], and [16]. The reported c.464G>A (p.Arg155Gln) [12,16] variant, novel variants c.1610G>A (p.Asp537Asn) and c.1643A>G (p.Asn548Ser) (Figure 1a, b) were found both in controls and patients with statistically insignificant association with the disease. Reported silent variants c.112G>A (p. =), c.1485G>A (p. =) [12] were also identified. The p values of these alleles showed significant association with the disease however the odds ratio (OR) was significantly lesser on comparing the disease with controls (Table 1).

**Table 1:** Allele and genotype frequencies of *COL8A2 *and *SLC4A11 *variants

Gene	Variation			FECD		Normal		P-value
				(n = 80)	%	(n = 100)	%	
***COL8A2***	c.112G>A (p. =)	Genotype	GG	67	83.8	93	93	0.104
			GA	9	11.3	6	6	
			AA	4	5	1	1	
		Allele	G	143	0.894	192	0.96	0.014
			A	17	0.106	8	0.04	0.014
			
	c.464G>A (Arg155Gln)	Genotype	GG	79	98.8	93	93	NS
			GA	0	0.0	5	5	
			AA	1	1.25	2	2	
		Allele	G	158	0.987	191	0.955	NS
			A	2	0.012	9	0.045	
			
	c.1485G>A (p. =)	Genotype	GG	62	77.5	91	91	0.028
			GA	13	16.3	8	8	
			AA	5	6.3	1	1	
		Allele	G	137	0.856	190	0.95	0.002
			A	23	0.144	10	0.05	0.002
		
	c.1610G>A (Asp537Asn)*	Genotype	GG	78	97.5	95	95	NS
			GA	2	2.5	5	5	
			AA	0	0.0	0	0.0	
		Allele	G	158	0.987	195	0.975	NS
			A	2	0.012	5	0.025	
		
	c.1643A>G (Asn548Ser)*	Genotype	AA	75	93.8	93	93	NS
			AG	5	6.3	7	7	
			GG	0	0.0	0	0.0	
		Allele	A	155	0.969	193	0.965	NS
			G	5	0.131	7	0.035	

***SLC4A11***	c.405G>A (p. =)	Genotype	GG	78	97.5	94	94	NS
			GA	2	25	6	6	
			AA	0	0.0	0	0.0	
		Allele	G	158	0.98	194	0.97	NS
			A	2	0.012	6	0.015	
		
	c.481A>C (p. =)	Genotype	AA	76	95	93	93	NS
			AC	4	5	7	7	
			CC	0	0.0	0	0.0	
		Allele	A	156	0.975	193	0.96	NS
			C	4	0.025	7	0.035	
		
	c.639G>A (p. =)	Genotype	GG	79	98.75	95	95	NS
			GA	1	1.25	5	5	
			AA	0	0.0	0	0.0	
		Allele	G	159	0.99	195	0.97	NS
			A	1	0.006	5	0.025	
		
	c.1659C>T (p. =) *	Genotype	CC	72	90	95	95	NS
			CT	8	10	5	5	
			TT	0	0.0	0	0.0	
		Allele	C	152	0.95	195	0.97	NS
			T	8	0.05	5	0.025	
		
	c.1974C>T (p. =) *	Genotype	CC	76	95	91	91	NS
			CT	4	5	9	9	
			TT	0	0.0	0	0	
		Allele	C	156	0.97	191	0.955	NS
			T	4	0.02	9	0.045	

The numbering is based on the complementary DNA sequence, with +1 corresponding to the A of the ATG translation initiation codon. All the variants identified both in *COL8A2 *and *SLC4A11 *were shown in this table. Asterisk (*) indicates novel variants identified in this study. NS means Not Significant. P value is considered to be statistically significant if P < 0.05.

*SLC4A11 *gene screening did not identify any pathogenic mutation. However 2 novel variants c.1659C>T (p. =), c.1974C>T (p. =) (Figure 1c, d) and 3 reported variants c.405G>A (p. =), c.481A>C (p. =), c.639G>A (p. =) [19] were identified. None of the variants were found to have any statistically significant association with the disease (Table 1). Moreover variants identified in *COL8A2 *and *SLC4A11 *did not seem to alter any splice site.

## Discussion

The exact cause of FECD is still unknown. To understand the pathogenesis, we screened for *COL8A2 *and *SLC4A11 *gene variants in our study. Previous studies in other ethnic groups identified pathogenic mutations in *COL8A2 *in association with FECD [12-16]. Biswas et al., [12] reported that p.Gln455Lys was detected in 3 early onset FECD families originating from Northern England and Australia. Also in their study, missense mutations p.Arg155Gln, p.Arg304Gln and p.Arg434His were identified in the common, late onset form of FECD. Similar to this report the heterozygous p.Gln455Lys mutation was also identified in Korean patients with FECD [16]. Gottsch et al., [13] reported the segregation of *COL8A2 *p.Leu450Trp with the disease phenotype in American familial FECD with early onset subtype. A study by Liskova et al., also observed the same mutation in a British family with early-onset FECD [15].

At birth, the normal endothelial cells start producing the posterior non-banded layer and continue to add material throughout life. In FECD and CHED2, Descemet membrane may appear thickened as the posterior layer is attenuated or absent and is replaced by an abnormal banded layer (varying from 14 to 20 μm, unlike the normal thickness 12 μm) due to endothelial dysfunction. This common clinical feature implies that *SLC4A11 *gene, the causative gene for CHED2 may play a role in FECD, even though the onset and symptoms of CHED2 and FECD are completely dissimilar. This is supported by the identification of heterozygous missense mutations in the *SLC4A11 *gene in Chinese and Indian FECD patients [19].

In the present study we did not identify any pathogenic mutations in *COL8A2 *in association with FECD. Even though association of c.112G>A (p. =) and c.1485G>A (p. =) was identified with FECD patients, these mutations may not be considered as pathogenic because they do not produce any change in the encoded amino acid and also do not alter the splice site. Similarly a study by Afshari et al., [10] did not identify p.Leu450Trp and p.Gln455Lys variants in their study of screening 92 FECD patients. Kobayashi et al., [22] and Mok et al., [16] observed p.Arg155Gln and p.Thr502Met variations in the *COL8A2 *gene but there were no statistically significant differences in the frequencies of these 2 variations between the affected individuals and control subjects. In other 4 independent studies no mutations were identified in *COL8A2 *in association with both early and late-onset FECD [23-26].

In *SLC4A11 *screening, we identified 2 novel and 3 reported silent variants which had no significant association with FECD and did not identify any pathogenic variants. By the screening of 25 Indian FECD cases Vithana et al., identified a presumed pathogenic variant c.1195G>A (p.Glu399Lys) in *SLC4A11 *in a single sporadic case. Though they found association of *SLC4A11 *with FECD, the mutations contribute to only 5% of the genetic burden of the disease [19].

## Conclusions

This is the first report analysing the *COL8A2 *gene in association with Indian FECD patients and we additionally studied the *SLC4A11 *gene in large number of Indian cohort. In the patients screened, we did not identify any pathogenic variants in both the genes making it unlikely that single nucleotide polymorphisms or mutations in them cause FECD. The possibility of pathogenic changes being within the promoter, intronic or untranslated non coding regions of these genes playing a role in the pathogenesis of FECD has not been excluded in this study.

Together with information from previous investigations, our study suggests that locus heterogeneity exist for FECD, wherein mutations of several genes on different chromosomes may produce a common disease phenotype. In addition to the genomic approach, proteonomic approach may also shed light on the disease pathogenesis.

## Competing interests

The authors declare that they have no competing interests.

## Authors' contributions

BH involved in acquisition of data, analysis and interpretation of data, statistical analysis and drafting of the manuscript. MS examined and recruited the cases, involved in planning the study and revising the manuscript. JAK examined and recruited the cases, involved in drafting of the manuscript and revising the manuscript. NVP examined and recruited the cases, involved in planning the study and revising the manuscript. PS designed the study and critically reviewed and modified the manuscript. All authors have read the final version of the manuscript and approved it for publication.

## Pre-publication history

The pre-publication history for this paper can be accessed here:

http://www.biomedcentral.com/1471-2415/10/3/prepub
